# A Working Hypothesis for the Role of the Cerebellum in Impulsivity and Compulsivity

**DOI:** 10.3389/fnbeh.2019.00099

**Published:** 2019-05-07

**Authors:** Marta Miquel, Saleem M. Nicola, Isis Gil-Miravet, Julian Guarque-Chabrera, Aitor Sanchez-Hernandez

**Affiliations:** ^1^Área de Psicobiología, School of Health Science, Universitat Jaume I, Castellón de la Plana, Spain; ^2^Dominick P. Purpura Department of Neuroscience, Albert Einstein College of Medicine, Bronx, NY, United States; ^3^Department of Psychiatry and Behavioral Sciences, Albert Einstein College of Medicine, Bronx, NY, United States

**Keywords:** cerebellum, compulsivity, addiction, impulsivity, prediction, habits

## Abstract

Growing evidence associates cerebellar abnormalities with several neuropsychiatric disorders in which compulsive symptomatology and impulsivity are part of the disease pattern. Symptomatology of autism, addiction, obsessive-compulsive (OCD), and attention deficit/hyperactivity (ADHD) disorders transcends the sphere of motor dysfunction and essentially entails integrative processes under control of prefrontal-thalamic-cerebellar loops. Patients with brain lesions affecting the cortico-striatum thalamic circuitry and the cerebellum indeed exhibit compulsive symptoms. Specifically, lesions of the posterior cerebellar vermis cause affective dysregulation and deficits in executive function. These deficits may be due to impairment of one of the main functions of the cerebellum, implementation of forward internal models of the environment. Actions that are independent of internal models may not be guided by predictive relationships or a mental representation of the goal. In this review article, we explain how this deficit might affect executive functions. Additionally, regionalized cerebellar lesions have been demonstrated to impair other brain functions such as the emergence of habits and behavioral inhibition, which are also altered in compulsive disorders. Similar to the infralimbic cortex, clinical studies and research in animal models suggest that the cerebellum is not required for learning goal-directed behaviors, but it is critical for habit formation. Despite this accumulating data, the role of the cerebellum in compulsive symptomatology and impulsivity is still a matter of discussion. Overall, findings point to a modulatory function of the cerebellum in terminating or initiating actions through regulation of the prefrontal cortices. Specifically, the cerebellum may be crucial for restraining ongoing actions when environmental conditions change by adjusting prefrontal activity in response to the new external and internal stimuli, thereby promoting flexible behavioral control. We elaborate on this explanatory framework and propose a working hypothesis for the involvement of the cerebellum in compulsive and impulsive endophenotypes.

## Introduction

Compulsivity and impulsivity have been proposed as neurocognitive endophenotypes for a heterogeneous group of mental disorders (Dalley et al., 2011; Robbins et al., 2012) such as addiction, eating disorders, attention deficit/hyperactivity (ADHD), obsessive-compulsive (OCD), as well as other personality and neurodevelopmental disorders. Endophenotypes reflect underlying predisposing factors for the vulnerability to psychopathology (Miller and Rockstroh, 2013). With respect to compulsivity and impulsivity, both endophenotypes involve a failure in top-down control and response inhibition but they do not always coexist in the same disorder (Dalley et al., 2011; Robbins et al., 2012). Compulsivity is characterized by an over-engagement in behavioral or cognitive activities despite their countless negative consequences. Compulsive behavior is persistent and inappropriate to the context entailing a failure in terminating actions properly (Robbins et al., 2012). Impulsivity is the trend to showing a premature and poorly planned behavior inappropriate to the context (Moeller et al., 2001). It can be expressed at the behavioral level as impulsive actions (difficulty in stopping an ongoing response) but also as impulsive choices (failure in delaying gratification; Robinson et al., 2009; Dalley et al., 2011). Compulsivity and impulsivity may contribute in varying degrees to the grounds of the disorder, which tends to be more severe when both endophenotypes occur together (Fineberg et al., 2014).

It has been proposed that cortico-striatal-networks mediate motor and cognitive domains for each construct (Dalley et al., 2011). Essentially, the proposed network dysregulation to explain top-down control deficits comprises an imbalance between dorsal and ventral zones in behavioral control, with an under-activation of the dorsal frontal regions along with an over-activation of striatal zones (Fineberg et al., 2010). None of the accepted neuroanatomic models have included the cerebellum, even though much data shows its involvement in different forms of impulsivity and compulsivity. Here, we review these scattered but consistent findings and synthesize a working hypothesis for the cerebellum’s contribution to impulsive and compulsive behavior.

## The Cerebellum: Too Many Neurons Just to Not Walk Like a Robot

The cerebellum includes 60 billion neurons, representing 80% of the total number of brain neurons (Azevedo et al., 2009; Barton, 2012; Lent et al., 2012). The reason why there are so many neurons in the cerebellum is one of the mysteries in brain evolution. Barton (2012) hypothesized that the cerebellum and cortico-cerebellar networks are fundamental components of the integrative brain systems enabling the prediction, organization, modeling and comprehension of complex sequences. This hypothesis is supported by evidence of cerebellar contributions to apparently dissociated brain functions that are altered in compulsive/impulsive disorders. These include Pavlovian conditioning (Pakaprot et al., 2009; Carbo-Gas et al., 2014a,b, 2017; Gao et al., 2016; Giovannucci et al., 2017); repetitive sequential learning (Wu et al., 2004; Doyon and Benali, 2005; Balsters and Ramnani, 2011); skill learning (Callu et al., 2007; De Bartolo et al., 2009); language (Leiner et al., 1993; Mariën et al., 2014; Verly et al., 2014); planning/prediction (Bastian, 2006; Bhanpuri et al., 2013); and social behavior (Kerney et al., 2017; Giocondo and Curcio, 2018; Hickey et al., 2018).

As established by tracing techniques, electrostimulation and optogenetics, the cerebellum appears to be closely connected to the functional loops that sustain compulsive and impulsive behavior (Ding et al., 2013; Herrera-Meza et al., 2014; Bostan and Strick, 2018). Thus, deep brain stimulation of the mediodorsal thalamic nuclei increases cFos expression in the deep cerebellar nuclei and the prefrontal cortex in rats (Moers-Hornikx et al., 2009). Additionally, cortical regulation of striatal activity can be modulated by the cerebellum (Moers-Hornikx et al., 2009). Furthermore, a direct dopaminergic VTA-cerebellar projection has also been demonstrated with detectable DA levels in the posterior lobules of the vermis (VII–X), the right and left hemispheres and the fastigial, interpositus and dentate nuclei (Glaser et al., 2006). More importantly, it has been shown that the cerebellar cortex may regulate dopamine release by several independent pathways. First, the cerebellum connects to the VTA through the reticulotegmental and pedunculopontine nuclei (Carbo-Gas et al., 2014b). Second, the cerebellum projects to the VTA through the mediodorsal and ventrolateral thalamus (Rogers et al., 2011). Finally, the deep cerebellar nuclei project directly to the VTA (Watabe-Uchida et al., 2012; Carta et al., 2019).

For some time now, it was proposed that the majority of mental disorders result from the dysregulation of normal central nervous system (CNS) development (Weinberger, 1987). Interestingly, the final cerebellar structure and functionality are developed postnatally, making cerebellar circuitry susceptible to alteration by external and internal factors at different developmental stages (Koziol et al., 2014). As an example, in terms of comparative anatomy, 50% of the cerebellum’s adult weight is achieved at birth in primates, at 15 postnatal days in rats and after 1 year in humans (Howard, 1973; Watson et al., 2006). Moreover, the cerebellar growth asymptote is reached in rats at 400 postnatal days (Sullivan et al., 2006) and around the age of 4 years in children (Dobbing and Sands, 1973). Alterations of the cerebellum during very early periods of postnatal life (prenatal, and neonatal) are able to shape morphology and functions of the mature brain (Limperopoulos et al., 2014). Several studies suggest that cerebellar injuries during perinatal/postnatal stages are sufficient to bring alterations in distal cortical regions (for a review, see Wang et al., 2014). For example, early cerebellar damage has been associated with a reduction in the modulation of dopamine release in the medial prefrontal cortex as well as the reorganization of cerebello-cortical loops (Rogers et al., 2011, 2013). In rodent models, compensatory responses and changes in the activity of brain-related regions have been found to be much greater when cerebellar injuries occurred within the neonatal period (Lalonde and Strazielle, 2015). In children, prenatal and neonatal lesions of the cerebellum generate motor dysfunction (Stoodley and Limperopoulos, 2016) but also cognitive and emotional deficits such as increased anxiety and aggressive behavior (Watson et al., 2018); autistic-like symptomatology in language and social behavior as well as selective attention deficits (Steinlin et al., 2003; Schmahmann et al., 2007). Adult lesions produce a more limited cortical compensation (O’Donoghue et al., 1986). Likewise, alterations in plasticity genes such as neuregulin 1, N-Methyl-D-aspartate (NMDA) and GABA signaling genes, which results in disruptions of the normal CNS development, accompany prefrontal-cerebellar pathology in schizophrenia and autism (Andreasen, 1999; Nopoulos et al., 1999; Allen et al., 2004; Scott et al., 2009; Yeganeh-Doost et al., 2011; Edmonson et al., 2014; Koziol et al., 2014; Murphy et al., 2014; Shevelkin et al., 2014; Osipowicz et al., 2015). Additionally, numerous neuroimaging studies have found structural abnormalities and changes in connectivity in the cerebellum of addicted cohorts (Barrós-Loscertales et al., 2011; Yu et al., 2011; Bora et al., 2012; Ersche et al., 2012; Ding et al., 2013; Koehler et al., 2013; Segobin et al., 2014; Shen et al., 2018) and members of high-risk families that did not take drugs (Hill et al., 2011).

In summary, prefrontal-cerebellar physiopathology is common in the comorbid mental disorders in which compulsive and impulsive endophenotypes are present. However, prefrontal-cerebellar alterations are not homogeneous and can affect different regions within these loops (for instance, dorsomedial prefrontal cortex vs. orbitofrontal cortex or the posterior vs. the anterior cerebellum), which might explain why prefrontal-cerebellar dysfunction is also implicated in many mental disorders in which compulsivity and impulsivity are not always key symptoms. These include schizophrenia (Andreasen, 1999; He et al., 2018) depressive disorders (Yucel et al., 2013; Scheinost et al., 2018; Wang et al., 2018) as well as fear and anxiety disorders (Richter et al., 2005; Picó-Pérez et al., 2017). Therefore, dysfunctional prefrontal-cerebellar loops do not always result in compulsivity and impulsivity though they generate a failure in top-down and executive control that in turn can bring compulsive and impulsive symptoms.

## Cerebellar Underpinnings of Compulsivity

The compulsivity construct is far from unitary (Fineberg et al., 2014; Figee et al., 2016). Several dissociable dimensions of compulsivity have been proposed, including cognitive inflexibility, motor disinhibition, disadvantageous decision-making, attentional bias, impaired executive planning and bias toward habits. Deficits in inhibition of motor responses do not always coexist with impaired cognitive flexibility. For instance, OCD patients show both impaired motor inhibition and clear deficits in cognitive flexibility, whereas other compulsive disorders such as trichotillomania appear to be limited to impaired inhibition of motor behaviors (Chamberlain et al., 2005, 2006). Thus, the dimensions of the compulsivity construct seem to represent different clusters of brain functions mediated by neuroanatomically and neurochemically distinct components of cortico-subcortical circuitry (Hollander et al., 2016). Overall, compulsivity implicates a failure in top-down cortical control that causes behavioral disinhibition. The resulting behavioral deficits may additionally be due to over-activity in the basal ganglia, which promotes automatic and stereotyped behavioral repetition (Fineberg et al., 2010).

In the next section, we describe the evidence for cerebellar changes in patients with compulsive disorders, exploring the consequences of alterations in the cerebellum for several of the described dimensions of compulsivity.

### Structural Neuroimaging Findings in the Cerebellum of Subjects Suffering From Compulsive Disorders

Untreated polydrug abusers exhibited decreased gray matter (GM) in the cerebellum and frontoparietal cortices along with increased GM in basal ganglia (Ersche et al., 2011). Additionally, GM volume in Crus I of the cerebellum was described to be correlated with the severity of nicotine dependence (Shen et al., 2018). Similar results were found in other compulsive pathologies such as internet gamblers (Dong et al., 2012; Ding et al., 2013); OCD (Ersche et al., 2011) as well as genetic disorders including compulsive symptoms such as Prader-Willi syndrome (Ogura et al., 2011). By contrast, greater cerebellar GM volume has been reported in healthy, non-drug-abusing members of families at high risk for alcohol dependence as compared to members of control low-risk families (Hill et al., 2011).

Regarding functional connectivity, OCD patients exhibit stronger interconnectivity between the cerebellum and the basal ganglia than the control subjects but weaker interconnectivity with the prefrontal cortex (Vaghi et al., 2017). As the authors indicated, these results suggest less top-down control over the prefrontal cortex on the lower regions.

Therefore, the most common structural findings in the cerebellum of patients with compulsive disorders have been decreased GM volume in several regions of the cerebellum and increased basal ganglia-cerebellar connectivity (Barrós-Loscertales et al., 2011; Ersche et al., 2011; Ogura et al., 2011; Yu et al., 2011; Dong et al., 2012; Ding et al., 2013; Segobin et al., 2014; Vaghi et al., 2017; Shen et al., 2018).

### Compulsivity and Prediction

Perhaps surprisingly, compulsivity may be due in part to deficits in the brain’s ability to make predictions. Some forms of decision-making require internal models of the environment that guide future choices using past outcomes (Blackwood et al., 2004). These internal representations, also called forward models, use memory to integrate predictions about the consequences of any given action with internal and external sensory inputs of the current state (Ito, 2008; Molinari et al., 2009). Individuals suffering from compulsive disorders demonstrate specific deficits in tasks in which behavioral outcome is regulated by internal models (mental representations of the world such as inferences). In a predictive-inference task, OCD patients do not consider the history of outcomes in order to regulate performance (Vaghi et al., 2017). They seem to have the capacity to establish the internal model but then the internal model fails to guide behavior. Vaghi et al. (2017) hypothesized that, in this case, actions become independent of internal models, leading to constant attempts to check the environment in order to adjust behavior. According to this suggestion, compulsive behavior entails a dysregulation of the integrative brain sensory-motor mechanisms that allow the use of predictive relationships to plan ahead and control behavior on line.

Accordingly, a role for the cerebellum in compulsive behavior is suggested by the extensive literature indicating that one of the main functions of the cerebellum is to implement internal models (Ito, 1990, 1993, 2008). An internal model is similar to a mental model but implicit (Ito, 2008). A mental model is a schematic representation of reality that is used to explain the present events and predict the future (Johnson-Lairds, 1983). The prefrontal cortex acts as a controller to create and manipulate the mental representations of the world that are distributed throughout the sensorimotor cortices. The cerebellar internal model works as an implicit and thereby unconscious template of this mental sensorimotor representation of the world. Then, the cerebellum processes the current functional state using sensory, interoceptive, and proprioceptive information. If there is a match between the mental model and bottom-up information (as can occur in overlearned tasks), the next event can be predicted from the template (Wolpert et al., 1998; Ito, 2008; Leggio and Molinari, 2015). In case of a discrepancy between “what I want to do” (prefrontal cortex) and “what is being done” (sensory-motor responses), the cerebellum generates an error signal that is essential for updating the internal model and making behavioral adjustments on line (Fautrelle et al., 2011).

If cerebellar error signaling fails (e.g., after cerebellar damage or dysfunction), one would expect internal models to fail to update and therefore to be unable to influence behavioral adjustments. Consistent with this hypothesis, cerebellar lesions impair this predictive capacity in motor tasks such as reaching. Patients with lesions cannot generate anticipatory adjustments and fail to make ongoing corrections reaching objects (Manto et al., 1995; Chen et al., 2006; Bhanpuri et al., 2013). We propose to extend the hypothesis to compulsive behavior in that cerebellar impairment could affect the ability to terminate a wide range of ongoing behaviors when environmental contingencies change. Indeed, clinical reports of patients with cerebellar disease or lesions demonstrate the emergence of compulsive arm shaking, checking, washing, and stereotyped motor activities (Gonzalez and Philpot, 1998). Clinical studies have also suggested decision-making deficits after cerebellar injury (Cardoso et al., 2014).

**Figure 1:**
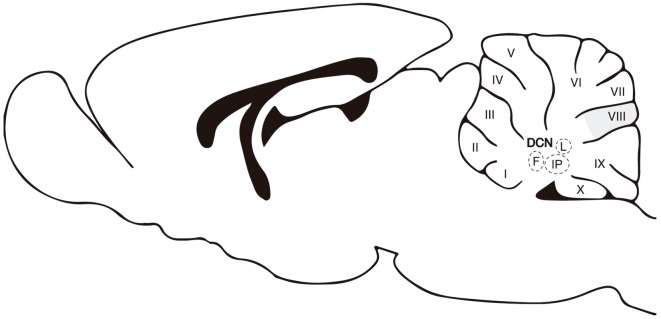
A sagittal view of the cerebellum. Abbreviations: DCN, deep cerebellar nuclei; M, medial or fastigial nucleus; IP, interpositus nucleus; L, lateral or dentate. Roman numbers correspond to lobules of the cerebellar cortex. The apical/dorsal region of lobule VIII is highlighted in gray.

In drug addiction, drug-related stimuli evoke drug memories and have the capacity to trigger craving and compulsive drug seeking (Shaham et al., 2003; Pickens et al., 2011). Importantly, neuroimaging studies of cue reactivity in drug addicts have consistently shown cerebellar activations when drug-related cues are presented (for a review, see Jasinska et al., 2014; Moulton et al., 2014; Miquel et al., 2016; and Moreno-Rius and Miquel, 2017). We used animal models to investigate the accurate location of the cerebellar area involved in these drug-cue associations (Carbo-Gas et al., 2014a,b, 2017). Our findings indicated that expression of cocaine-induced conditioned memory is accompanied by a selective increase in neural activity at the most external part of the granular cell layer in the posterior cerebellum (Figure 1). Such increase was only seen in animals that acquired the memory, but not in pseudo-conditioned groups or in animals that, despite being trained in a contingent association, did not express the conditioned response towards cocaine-related cues (CS+). More importantly, this cerebellar activity appeared to be one of the correlates of the behavioral decision driven by the drug-related cue. Accordingly, when animals were confined in the presence of the CS+ with no opportunity to select other behavioral alternatives, cerebellar activity was normalized to control levels (Carbo-Gas et al., 2017). Recently, we proposed that the cerebellar cortex biases behavioral selection towards the context that predicts drug availability, and that this happens by generating predictions after presentation of the conditioned cue (Carbo-Gas et al., 2014b; Moreno-Rius and Miquel, 2017). We propose that the internal state modulates these predictions, increasing the probability of selecting the drug-associated context when the drug is absent in the body. In this way, the cerebellum, by activating drug-cue representations during abstinence, can contribute to compulsive drug seeking driven by both negative and positive reinforcement.

### Compulsivity, Habit Formation and Executive Behavioral Control

Classically, habits have been considered as overlearned, repetitive, sequential behaviors that are performed automatically and triggered by associated environmental signals (Graybiel, 2008). During acquisition of habits, there is a shift from goal-directed behavior regulated by an action-outcome process (R-O) to automatized responses triggered by the stimuli (S-R; Dickinson and Weiskrantz, 1985). Nevertheless, as Robbins and Costa (2017) have discussed recently, habits and skills are not equivalent processes. Habits refer to “which stimuli elicit the behavior” and do not necessarily involve overtraining. Like goal-directed behavior, habits are “autonomous from the goal” and thus outcome devaluation is unable to reduce the presence of habitual behavior. However, skills involve sequential learning that requires extended training though they may be goal-directed, and thereby still dependent on the outcome. Three dissociated but interconnected loops including different cortical and striatal regions have been proposed to underlie and control the establishment of habits: the limbic, associative and sensorimotor networks (Yin and Knowlton, 2006).

Neuroimaging studies of skill learning reported cerebellar deactivations during the automatic phase (Wu et al., 2004; Doyon and Benali, 2005; Balsters and Ramnani, 2011). Both the prefrontal cortex and cerebellum decrease their activity as sequential learning progresses. Then, if task demands increase, prefrontal cortex activity is engaged again but the cerebellum remains deactivated (Doyon and Benali, 2005). Electrophysiological recordings performed in the cerebellar cortex of rodents during motor learning showed similar findings. In these studies, the initial learning phase was characterized by high cerebellar cortical activity, which decreases with trials and repetition (de Zeeuw and Yeo, 2005; Garcia-Martinez et al., 2010). Thus, correlational research on the cerebellar contribution to motor learning suggested that the prefrontal cortex and cerebellum work in parallel during acquisition and progression of learning, but they are recruited differently when cognitive and motor demands grow. In accordance, non-invasive stimulation of the cerebellum supports the role of the cerebellum in the initial phase of motor learning (Darch et al., 2018). By contrast, hemicerebellectomy seems to delay the transition to response automatization rather than impair acquisition of sequential learning (Mandolesi et al., 2010).

Additional evidence strongly supports the contribution of the cerebellum to habits. During instrumental actions, goal-directed behavior (R-O) can compete with the stimulus-response automatic mechanism (S-R). When habitual behavior is established, the probability of responding for devalued outcomes increases (Adams and Dickinson, 1981). The ability to resolve and monitor the competition between habit and goal-directed processes depends on the engagement of inhibitory executive control (Watson et al., 2018). Contrary to the frontal pole, the cerebellum and other regions in the sensorimotor network, such as the premotor cortex, show greater activation when subjects respond to previously devalued outcomes, suggesting that they participate in the expression of S-R habits (Watson et al., 2018). Accordingly, in an elegant study, Liljeholm et al. (2015) demonstrated using functional imaging in humans that neuronal activity in the tail of the caudate/thalamus, the cerebellum and the lingual gyrus predicts insensitivity to devaluation. Participants with greater activity within these regions in the S-R relative to R-O conditions during the two first blocks of the instrumental learning phase responded to a greater proportion of trials with devaluated outcomes during the test phase. Of particular relevance is the fact that in Liljeholm’s study the formation of habit did not require overtraining because R-O and S-R were trained separately as different experimental conditions for the same number of trials. Thus, activity within the caudate and cerebellum was not a function of practice or repetition, but rather it predicted the formation of a strong stimulus-response association. This observation suggests a significant role not only of the basal ganglia but also the cerebellum in S-R habit formation.

Consistent with the human imaging studies, impaired ability to inhibit responding to the previously-rewarded but no-longer-correct stimulus (perseverative errors) has been observed both in a rodent model of autism in which cerebellar dysfunction is present (Dickson et al., 2010) and in hemicerebellectomized rats (De Bartolo et al., 2009). Moreover, a bilateral lesion in the interpositus nucleus of the cerebellum prevents rats from developing habits with overtraining (Callu et al., 2007). In these rats, behavior maintains the action-outcome features and transition to the automatic cue-response stage is not created.

Therefore, the cerebellum is not critical to learning goal-directed behaviors but appears to be required for habit and skill learning. Furthermore, lesion studies suggest that the integrity of the cerebellum is essential for the brain process underlying habit formation. Future research will ascertain whether the cerebellum is essential to habit formation or instead to the expression of habits.

Similar to the interpositus, the infralimbic prefrontal cortex has been demonstrated to be crucial for the establishment of habits in that repeated optogenetic inhibition of the infralimbic cortex disrupts habit formation (Smith and Graybiel, 2013). Impairment of the infralimbic cortex suppressed the shift away from goal-directed behavior to habitual reward seeking, even after substantial overtraining (Killcross and Coutureau, 2003; Miles et al., 2003; Smith and Graybiel, 2013).

It has been hypothesized that compulsive behavior may result from aberrant habit formation (Everitt and Robbins, 2005). In fact, patients suffering from compulsive disorders including OCD, drug addiction, and Tourette syndrome develop habits more easily than controls after reduced behavioral training (Gillan et al., 2011; Hogarth and Chase, 2011; Delorme et al., 2016; Ersche et al., 2016). According to one theory of drug addiction, compulsivity characterizes late stages of the disorder (Everitt and Robbins, 2005; Koob and Volkow, 2010; Montigny et al., 2013). In the initial stages of drug intake, drug-related cues drive goal-directed behaviors towards contexts with drug availability. With extended drug experience, cue-action-outcome relationships can become over-consolidated, and drug-related cue/context can activate automatic behaviors (Everitt and Robbins, 2005).

The contribution of the cerebellum to the transition from recreational drug intake (goal-directed behavior) to compulsive habits is still unknown, although human and animal neuroimaging research supports a reorganization of the prefrontal-cerebellar network in addicted patients (Hester and Garavan, 2004; Bolla et al., 2005; Goldstein et al., 2007) and other primates with a history of cocaine self-administration (Porter et al., 2014). Altogether, these findings indicate that the downregulation in prefrontal cortices during addiction is accompanied by abnormal greater activity in the cerebellum when task demands increase. Importantly, the sensorimotor network, including brain regions underlying motor skills learning and action, increase their activity when drug-related cues are presented (Yalachkov et al., 2009). Thus, smokers showed higher activation than non-smokers in the right lateral cerebellum, the left premotor cortex, and the left superior parietal lobule during the presentation of smoking-related cues (Yalachkov et al., 2009). Additionally, smokers show more restrictive brain activity patterns than non-smokers during reward tasks. During a pattern-recognition task, a nonmonetary reward elicited activation only in the smokers’ cerebellum. In non-smokers, the brain pattern was wider involving the striatum, prefrontal cortex and limbic cortices. Moreover, the presentation of a monetary reward was unable to activate the striatum in smokers as compared to nonsmokers (Martin-Sölch et al., 2001).

**Figure 2:**
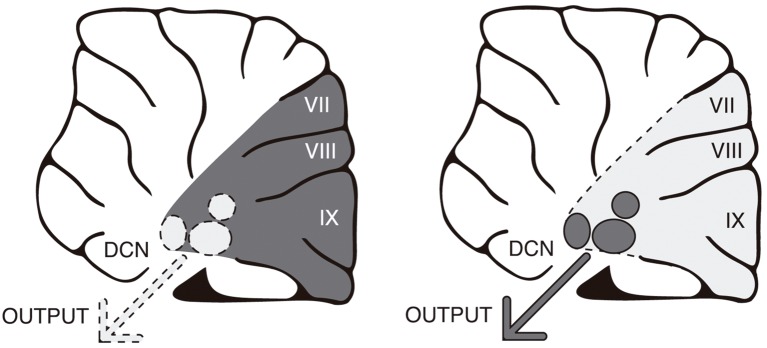
The cerebellar cortex tonically inhibits the deep nuclei through GABAergic Purkinje axons. In accordance, by reducing synaptic function in Purkinje neurons, it is possible to increase neuronal activity in the deep nuclei. Changes in activity are represented by dark gray (greater) vs. faint gray (lower).

Recent findings from our laboratory indicate that the cerebellum can control learning-related activity (Gil-Miravet et al., 2018) and plasticity in the infralimbic cortex (unpublished results). Neurotoxic lesion of the posterior cerebellar cortex performed before conditioning increased cFos expression and mechanisms for synaptic stabilization in the infralimbic cortex. It is plausible that the cerebellar cortex could influence infralimbic function through disinhibition of the deep cerebellar nuclei. The cerebellar cortex exerts an inhibitory tonic control over the deep nuclei through GABAergic Purkinje axons (Gauck and Jaeger, 2000; Figure 2). In accordance, by reducing the synaptic function in Purkinje neurons it is possible to increase neuronal activity and PNN expression in the deep nuclei (Vazquez-Sanroman et al., 2015). Overall, our results suggest that the cerebellar cortex may regulate infralimbic activity in an inhibitory manner *via* inhibition of the deep cerebellar nuclei. In this way, cerebellar dysfunction might contribute to the establishment of drug-induced incentive habits by controlling activity and plasticity in the infralimbic cortex.

In summary, the findings described above highlight the cerebellar role in compulsivity. First, a dysfunctional prefrontal-cerebellum network might mediate the inability to use internal models to regulate behavior. Second, the cerebellum is likely required for the formation and expression of habits. Third, in drug addicts and heavy drug users, impairment of executive functions has been repeatedly associated with a dysfunctional prefrontal-cerebellar pattern in which the cerebellum is overactive. Several years ago, we hypothesized that as the prefrontal cortex is downregulated by the repetition of drug experience, the cerebellum will increase its functional relevance, encouraging faster and automatic forms of control at the expense of behavioral flexibility (Miquel et al., 2009). So far, this hypothesis remains untested but it should be reformulated in light of the present evidence (see section “A Working Hypothesis for the Role of the Cerebellum in Compulsivity and Impulsivity”).

## Cerebellar Dysfunction in Impulsivity

Several dimensions contribute to motor and cognitive impulsivity including rapid decision making, intolerance to delays in reward delivery, as well as tendency to prematurely terminate response chains (Evenden, 1998). Separate cortico-striatal networks control different aspects of impulsivity (Eagle et al., 2008). The stop circuit involved in motor impulsivity comprises the right inferior frontal gyrus, anterior cingulate cortex, presupplementary and motor cortices, dorsal striatum (caudate/putamen), and subthalamic nucleus. Impulsive choices are triggered from the nucleus accumbens core, basolateral amygdala and orbitofrontal cortex. As with compulsivity, the cerebellum has been overlooked in the more influential anatomical models of impulsivity even though cerebellar dysfunction has repeatedly been linked to impulsive symptomatology (Mulder et al., 2008; Durston et al., 2011; de Zeeuw et al., 2013).

### Structural Neuroimaging in the Cerebellum and Impulsivity Disorders

Dysregulation of the cerebellum, particularly of the cerebellar vermis, has been accepted as a potential etiological component of ADHD (Mulder et al., 2008; Durston et al., 2011; de Zeeuw et al., 2013; Pieterman et al., 2018). Earlier structural neuroimaging studies with children and adults with ADHD described reduced cerebellar volumes even after correction for total cerebral volume (Berquin et al., 1998; Mostofsky et al., 1998; Castellanos et al., 2001). Similar to ADHD patients, smaller cerebellar volume and reduced GM have been observed in preterm children with impulsive symptomatology (Matthews et al., 2018); compared with normal term infants, preterm children are more likely to show impulsive behavior, inattention, cognitive inflexibility, and meet a diagnosis of ADHD (Foulder-Hughes and Cooke, 2007; Farooqi et al., 2013; Morales et al., 2013; Pozzetti et al., 2014; Franz et al., 2018). Likely, the dynamic of cerebellar development in the last trimester of gestation makes the cerebellum more vulnerable to dysfunction in a preterm birth than other brain regions (Tran et al., 2017).

More recent studies have gone further in identifying the different dimensions of impulsivity related to specific structural cerebellar abnormalities. For example, greater GM volume in the right cerebellum was associated with higher motor impulsivity levels (Lee et al., 2011). Impulsivity is not always dysfunctional and indeed it can be observed in non-pathological individuals as a predisposition to premature and poorly planned responses (Moeller et al., 2001). Interestingly, GM abnormalities in the cerebellum only correlate with dysfunctional impulsivity since high impulsivity in normal subjects involved a different pattern of GM correlations (Hogarth, 2011).

Additionally, abnormal cerebellar connectivity patterns have been described in ADHD patients. In these patients, the cerebellum exhibits reduced connectivity with the prefrontal cortex (Wolf et al., 2009). Oldehinkel et al. (2016) investigated striatal connectivity in an extensive sample of subjects with a diagnosis of ADHD as well as in their healthy relatives. Hyperactivity, impulsivity and inattention were related to greater connectivity of the posterior putamen with the cerebellum and occipital cortex.

### Impulsivity and Executive Function

Evidence for functional changes in the cerebellum of ADHD patients indicated attenuated cerebellar activity during the performance of executive tasks (Schulz et al., 2004; Valera et al., 2005). Neurofunctional models of ADHD have distinguished several subtypes of ADHD patients as a function of underlying brain pathways and primary functional deficits associated with them (Sonuga-Barke et al., 2008; Durston et al., 2011). The strongest evidence suggests an executive vs. reward-related dysfunction. In the first subgroup, patients show an impairment in behavioral inhibition including inattention (executive deficits). In the second one, the primary deficit was emotional/motivational and it was expressed as an aversion to delayed reward delivery (Sonuga-Barke et al., 2008; Durston et al., 2011). Inability to engage the cerebellum as well as prefrontal and parietal cortices during response inhibition tasks was found to be the hallmark for the subgroup with executive deficits (Stevens et al., 2018). Moreover, those patients with emotional and motivational-related deficits over-engaged the amygdala and ventral striatum during rewarded tasks with no change in prefrontal-cerebellar network.

Impulsivity is also present in bipolar disorder, the neuropsychopathology of which includes both executive and emotional-motivational deficits. In contrast to subjects diagnosed with ADHD, the difficulty in inhibiting a prepotent motor response in bipolar patients was accompanied by reduced striatal activity along with increased activation of the orbitofrontal cortex, amygdala and cerebellum (Fleck et al., 2011). Therefore, although the most common cerebellar correlate of behavioral disinhibition is reduced activity in the cerebellum, different pre-existing pathological conditions may constrain the type of brain pattern that will be observed during behavioral inhibition tasks.

In a recent genetic mouse model of ADHD (High-Active mice), downregulation of the prefrontal cortex was accompanied by hyperactivity in the granule cell layer of the cerebellar vermis during the performance of a high-speed rotarod task (Majdak et al., 2016). A low amphetamine dose normalized motor impulsivity symptom to control levels. However, amphetamine treatment reduced only cerebellar hyperactivity, leaving prefrontal downregulation unaltered. This finding points to the cerebellum as a therapeutic target for impulsive disorders similar to what has been suggested by studies using cognitive training in ADHD children (Hoekzema et al., 2010).

In drug abuse, impulsivity may act as a vulnerability factor to compulsive drug-seeking but also can be the result of repeated drug intake (Belin et al., 2008; Verdejo-García et al., 2008; Ersche et al., 2010; Hogarth, 2011; Whelan et al., 2012; Irimia et al., 2015). Cerebellar dysfunction has been proposed as one of the main factors to explain comorbidity between drug addiction and other impulsive disorders (Jasinska et al., 2014; Moulton et al., 2014; Miquel et al., 2016). Nevertheless, only a few studies have specifically investigated the cerebellar underpinnings of drug-related impulsivity. In alcoholic patients at different stages of remission, frontocerebellar dysfunction appears to be a key factor to predict and explain impulsive control deficits (Sullivan, 2003; Jung et al., 2014). Functional connectivity research demonstrated that anterior cingulate-cerebellar synchrony is degraded in alcoholics when responses have to be inhibited to avoid errors (Jung et al., 2014). Under uncertainty, alcoholics failed to activate the cerebellum, emitting more erroneous responses while compensatory activity was observed in the dorsal prefrontal and premotor cortices (Jung et al., 2014). Unlike alcoholics, adolescent cannabis users showed an increased correlation in the activity of the frontal-parietal-cerebellar network associated with poor inhibitory behavioral control in a Go/No-Go task (Behan et al., 2014). Greater correlation between the parietal cortex and cerebellum was also seen during resting state in cannabis users relative to control subjects. In this study, frontal-parietal-cerebellar hyper-connectivity did not compensate for performance as cannabis abuser committed more errors than the control group. Overall, despite the fact that both the type of drug and task conditions might be important factors for understanding the involvement of the cerebellum in drug-related impulsivity, aberrant cerebellar connectivity patterns are common to impulsive behavior in heavy drug users.

### Effects of Cerebellar Lesions on Impulsivity

Clinical reports on cerebellar diseases give support to the fundamental role of the cerebellum in modulating diverse motor, affective and cognitive domains. Beyond motor dysfunction, patients with lesions or disease affecting the posterior cerebellum showed difficulties in controlling their behavior and emotions, language deficits, and lack of concentration (Silveri et al., 1994; Schmahmann and Sherman, 1998; Kim et al., 2013; Tessier et al., 2015). The syndrome, which has been called “the cerebellar cognitive-affective syndrome,” is characterized by impairments in executive functions with disinhibited and inappropriate behavior, social aberrant behavior, personality changes, and language deficits (Schmahmann and Sherman, 1998).

In accordance with clinical observations, lesion studies in animals have established the relevance of the cerebellum for perseverative behavior and behavioral disinhibition (Bobée et al., 2000). Posterior vermis lesions result in a delay in behavioral inhibition during extinction trials (Callu et al., 2007). Animals that received vermis lesions when young showed perseverative behavior as adults, lack of attention to novel stimuli, and behavioral disinhibition (Bobée et al., 2000). Taken together, these results indicate that the cerebellum is a crucial component of the circuits controlling the inhibitory mechanisms for initiating actions.

## A Working Hypothesis for the Role of the Cerebellum in Compulsivity and Impulsivity

**Figure 3:**
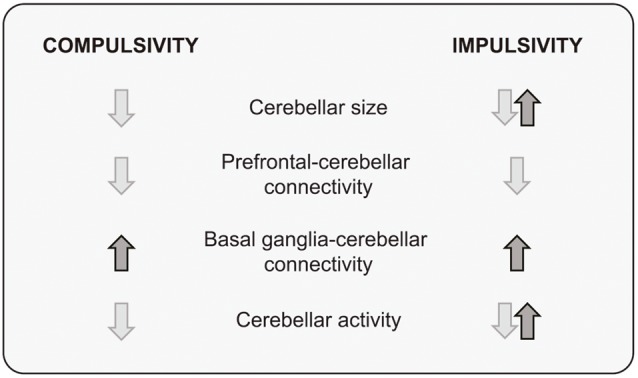
A summary of structural and functional cerebellar findings in compulsivity and impulsivity.

Although in many cases the evidence is incomplete and partial, a picture is beginning to emerge from research on the cerebellar contribution to compulsivity and impulsivity: pre- and postnatal developmental events can induce cerebellar dysfunction or alter cerebellar connectivity patterns, encouraging basal ganglia-cerebellum connectivity while degrading prefrontal-cerebellum connections. Thus, it appears that a consequence of disrupting cerebellar function is an imbalance between dorsal (downregulation) and ventral (upregulation) influences on behavior, facilitating an over-reliance of “Go” brain mechanisms at the expense of “No-Go” inhibitory control, with actions becoming persistent and inappropriate to the context (Figure 3).

Overall, findings point to a modulatory function of the cerebellum in terminating or initiating actions through regulation of the prefrontal cortices. That is, the cerebellum may be crucial for restraining ongoing actions when environmental conditions change by adjusting prefrontal activity in response to the new external and internal constellation of stimuli, thereby promoting flexible behavioral control. Both electrical and non-invasive stimulation of cerebellar activity in animals and humans support a modulatory effect of the cerebellum on cortical activity (Forster and Blaha, 2003; Chen et al., 2014; Watson et al., 2014). It has been hypothesized that the cerebellar modulation consists of what has been called “cerebellar brain inhibitory function” (Darch et al., 2018). If this is the case, one should expect stimulation of cerebellar activity to improve prefrontal functionality and to reduce compulsive and impulsive behaviors (Figure 2). Notwithstanding, cerebellar stimulation can reduce or increase cortical activity as a function of the stimulation protocol (Casula et al., 2016) as well as the cortical population targeted (Watson et al., 2014). Moreover, cerebellar modulation involves subtle changes in synchronization of cortical firing more than global changes in neuronal activity (Watson et al., 2014). Also relevant is the fact that the cerebellum is not a functional unit and therefore it should not be expected that manipulations across different regions of the cerebellum should produce homogeneous effects either on behavior or on brain activity. For instance, stimulation of the cerebellar cortex should result in opposite effects to stimulation of deep cerebellar nuclei as they receive tonic inhibitory GABAergic control through Purkinje cells (Gauck and Jaeger, 2000).

A comprehensive understanding of the cerebellar function in compulsivity and impulsivity will require further research involving causative manipulation of the cerebellar activity and its connectivity since the majority of the current information comes from correlational research and clinical reports. For instance, it is known that impulsivity and disinhibition result from impairment of the cerebellar cortex, especially in the middle line (vermis; Silveri et al., 1994; Schmahmann and Sherman, 1998; Kim et al., 2013; Tessier et al., 2015). Thus, it should be possible to interfere with or mimic these effects by using pharmacogenetics tools as DREADDs (designer receptor exclusively activated by designer drugs) or optogenetics in paradigms such as Go/No-Go tasks and reward devaluation tests. These research tools could also be applied to drug-related compulsivity and impulsivity in animal models of addiction such as those proposed by Vanderschuren and Everitt (2004); Ahmed (2012) or Deroche-Gamonet and Piazza (2014). Importantly, the specific contribution of the cerebellum to drug addiction is an almost utterly uncharted field. The present model (Figure 2) predicts that by inhibiting activity in the cerebellar cortex impulsive and compulsive symptomatology would increase. On the contrary, the stimulation of the cerebellar cortex should improve behavioral inhibitory control in the above-mentioned paradigms and models. Opposite predictions may be made for the effects of direct manipulations in the deep cerebellar nuclei (DCN), as the DCN receives tonic inhibition from the cerebellar cortex (Gauck and Jaeger, 2000). If confirmed our expectations, the cerebellum would appear as the next therapeutic target for impulsive/compulsive disorders.

## Author Contributions

Several of the results revised here were obtained by IG-M, JG-C and AS-H as a part of their doctoral theses. They also critically reviewed the content of the manuscript. MM is responsible for the hypothesis and wrote the review. SN critically read and edited the review. All the authors approved the final version for publication.

## Conflict of Interest Statement

The authors declare that the research was conducted in the absence of any commercial or financial relationships that could be construed as a potential conflict of interest.
